# A Proposal to Reflect Survival Difference and Modify the Staging System for Lung Adenocarcinoma and Squamous Cell Carcinoma: Based on the Machine Learning

**DOI:** 10.3389/fonc.2019.00771

**Published:** 2019-08-14

**Authors:** Ming Li, Cheng Zhan, Xizhao Sui, Wei Jiang, Yu Shi, Xiaodong Yang, Mingxiang Feng, Jun Wang, Qun Wang

**Affiliations:** ^1^Department of Thoracic Surgery, Zhongshan Hospital, Fudan University, Shanghai, China; ^2^Department of Thoracic Surgery, People's Hospital, Peking University, Beijing, China

**Keywords:** modification, TNM staging system, non-small cell lung cancer, survival difference, machine learning

## Abstract

**Objective:** To propose modifications to refine prognostication over anatomic extent of the current tumor, node, and metastasis (TNM) staging system of non-small cell lung cancer (NSCLC) for a better distinction, and reflect survival differences of lung adenocarcinoma and squamous cell carcinoma.

**Study Design:** Three large cohorts were included in this study. The training cohort consisted of 124,788 patients in the Surveillance, Epidemiology, and End Results (SEER) database (2006–2015). The validation cohort consisted of 4,247 patients from the Zhongshan Hospital, Fudan University (FDZSH; 2005–2014), and People's Hospital, Peking University (PKUPH; 2000–2017). The algorithm generated a hierarchical clustering model based on the unsupervised learning for survival data using Kaplan-Meier curves and log-rank test statistics for recursive partitioning and selection of the principal groupings.

**Results:** In the modified staging system, adenocarcinoma cases are usually at a lower stage than the squamous cell carcinoma cases of the same TNM, reflecting a better outcome of adenocarcinoma than that of squamous cell carcinoma. The C-index of the modified staging system was significantly superior to that of the staging system [SEER cohort: 0.722, 95% CI, (0.721–0.723) vs. 0.643, 95% CI, (0.640–0.647); FDZSH cohort: 0.720, 95% CI, (0.709–0.731) vs. 0.519, 95% CI, (0.450–0.586); and PKUPH cohort: 0.730, 95% CI, (0.705–0.735) vs. 0.728, 95% CI, (0.703–0.753)].

**Conclusion:** Survival differences between lung adenocarcinoma and squamous cell carcinoma have been reflected accurately and reliably in the modified staging system based on the machine learning. It may refine prognostication over anatomic extent.

## Introduction

Non-small cell lung cancer (NSCLC) is one of the most commonly diagnosed and leading causes of cancer death among both men and women worldwide (1–3). The survival duration underscores the importance of an accurate method to properly predict the prognoses of NSCLC patients to better manage this disease. The American Joint Committee on Cancer (AJCC) and the Union for International Cancer Control (UICC) staging systems of lung cancer using tumor, node, and metastasis (TNM) classification at the time of diagnosis and management, is the most frequently used predictor of survival and indicator of therapeutic strategies planning for NSCLC. The 8th edition of the TNM staging system of NSCLC was published by the International Association for the Study of Lung Cancer (IASLC) in January 2017, and it has been recommended to replace the 7th version (4). Modifying, the 8th TNM classification system for newly NSCLC introduced changes to the classification in both the T and M categories, as well as in the overall stage grouping (4). The upgrading and updating improved the discriminatory ability between adjacent subgroups. However, a heterogeneous aggregate of survival of adenocarcinoma and squamous cell carcinoma has not been discriminated like staging system of esophageal cancer. Recently, several studies have suggested that different prognoses may exist in patients with the same stage of adenocarcinoma and squamous cell carcinoma (5–8). Importantly, the difference between squamous cell carcinoma and adenocarcinoma in prognosis and survival has been evaluated in many studies and should not be ignored in a staging system when developing a more accurate discriminatory ability and prognostic performance in clinical practice (9–11); especially, the prognoses of some patients in the same sub-stage are different, when some cases with different sub-stages have similar prognoses. To further solve these problems, we aimed to propose modifications to refine prognostication over anatomic extent of the current TNM staging system for NSCLC, by considering the heterogeneity of survival and basing on the machine learning method.

## Methods

### Selection and Description of Participants

The training cohort of patients with NSCLC was from the Surveillance, Epidemiology, and End Results (SEER) database (2006–2015) of the National Cancer Institute. Only patients with microscopically confirmed squamous cell carcinoma or adenocarcinoma (ICO-O-3 histology/behavior codes 8,050–8,089 and 8,140–8,389, respectively) were included (12). Patients with other variants of lung cancer, such as large cell carcinoma and small cell lung cancer, were excluded. Patients without follow-up information were excluded. Patients who received chemotherapy before surgery (yp cases), or underwent resection for a recurrent lung cancer (r-stage cases) were not considered. All patients included in this study were artificially restaged according to the definitions of the 8th TNM staging system, based on the available clinical and pathological data, both in the SEER database and two validation cohorts.

The validation cohort of NSCLC patients was from the Department of Thoracic Surgery, Zhongshan Hospital, Fudan University (FDZSH; 2006–2015) and the Department of Thoracic Surgery, Peking University People's Hospital (PKUPH; 2000–2017). All patients received surgical treatment alone or combined with chemotherapy alone or with radiotherapy. In this study, there were no human subjects involved and only de-identified data were used, thus, ethical review and informed consent were waived by the institutional review board of Zhongshan Hospital, Fudan University. For the analysis of TNM categories presented, all patients were identified via histological and pathology diagnoses of NSCLC and cases with missing staging information or survival status were excluded. Patients were examined every 6 months during the first 2 years and annually thereafter. A physical examination, chest computed tomography scan, and abdominal ultrasound were included in the follow-up protocol. Bone scintigraphy and brain magnetic resonance imaging were performed when relative symptoms appeared.

### Statistics

Cancer specific survival (CSS) was defined as the period from the day of diagnosis to the day of death specified by the cancer or related complications. Survival duration was measured from the date of initial diagnosis for clinically staged tumors and from the date of surgery for pathologically staged tumors until the date of death due to the cancer or the date of the last follow-up and calculated by the Kaplan-Meier method. The algorithm generates a hierarchical clustering model based on the unsupervised learning for survival data using the distance matrix of survival curves, which calculated by the χ^2^ value of log-rank test with the assumption of patients of each group were equal and infinite, for recursive partitioning and selection of the principal groupings (13) (https://cran.r-project.org/web/views/Cluster.html). The calculation formula was as follows and the relevant values are calculated. For each time *i*, let a_i_ and b_*i*_ be the accumulative survival rate at the period *i* after diagnosis in the two groups, respectively.

$$\chi^{2}=\frac{\sum [a_{i-1}-a_{i}-a_{i}.\frac{\left(a_{i-1}-a_{i}+b_{i-1}-b_{i}\right)}{a_{i-1}+b_{i-1}}}{\sum \left[a_{i-1}.b_{i-1}.\frac{\left(a_{i-1}-a_{i}+b_{i-1}-b_{i}\right)}{\left(a_{i-1}+b_{i-1}\right).\left(a_{i}+b_{i}\right)}\right]}$$

The concordance index (C-index) was used to assess the discriminatory powers of the two staging systems, and the survival calibration curve was calculated to evaluate the calibration of the 8th IASLC staging system and the modified system (14, 15).

The analysis was implemented using the statistical package R, version 3.4.3 (R Project for Statistical Computing, TUNA Team, Tsinghua University) and Graphpad Prism 7 (GraphPad Software, Inc., San Diego, CA). A *p* < 0.05 was statistically significant, and all tests were two-sided.

## Results

### Characteristics of Patients

Overall, 124,788 patients, 1,675 patients, and 2,572 patients from the SEER database, FDZSH database, and PKUPH database with pathologically confirmed NSCLC were included in this study, respectively. Numbers of patients from the SEER cohort in stage IA to IV were 27,193; 7,066; 4,199; 10,512; 16,633; 9,092; 1,465; and 48,619, respectively. In the FDZSH and PKUPH cohorts, numbers of patients in stage IA to IV were 830, 155, 61, 243, 302, 68, 2, 14, and 1,052; 592, 76, 284, 395, 87, 2, 84, respectively. The baseline data of clinical and histopathological characteristics are shown in Table 1. In the SEER, FDZSH, and PKUPH cohorts, the proportion of male patients was higher than that of female patients. Consistently, most patients had tumors located at the upper lobe, and there were similar proportions of patients having adenocarcinoma and squamous cell carcinoma in the SEER, FDZSH, and PKUPH cohorts. More than half of the patients in the SEER cohort had moderately differentiated or poorly differentiated tumors. At the same time, the differentiation of the tumors in the FDZSH and PKUPH cohorts was similar to that in the SEER cohort. The 3-year CSS rate of the SEER cohort was 36.7% and the 5-year CSS rate was 29.1%. The 3-year CSS and 5-year CSS rates of the FDZSH s and PKUPH cohorts were 79.0 and 69.2%, and 82.8 and 74.0%, respectively.

**Table 1:** Clinical and histopathologic characteristics of patients.

**Characteristics**	**SEER Cohort** **(2006–2015) (*n* = 124,788)**	**FDZSH Cohort** **(2009–2014) (*n* = 1,675)**	**PKUPH Cohort** **(2000–2017) (*n* = 2,572)**
Gender			
Male	64,448 (51.6)	918 (54.8)	1,391 (54.1)
Female	60,340 (48.4)	757 (45.2)	1,181 (45.9)
Age, years			
<65	46,348 (37.1)	1,149 (68.6)	1,471 (57.2)
≥65	78,440 (62.9)	526 (31.4)	1,101 (42.8)
Location			
Upper lobe	75,130 (60.2)	882 (52.7)	1,313 (51.0)
Middle lobe	5,888 (4.7)	114 (6.8)	174 (6.8)
Lower lobe	37,072 (29.7)	518 (30.9)	763 (29.7)
Overlapping lesion of lung	1,396 (1.1)	155 (9.2)	133 (5.2)
Unknown	5,302 (4.3)	6 (0.4)	189 (7.3)
Histology			
Squamous cell carcinoma	42,380 (34.0)	434 (25.9)	497 (19.3)
Adenocarcinoma	82,408 (66.0)	1,241 (74.1)	2,075 (80.7)
Grade			
Well-differentiated; Grade I	9,730 (7.8)	34 (2.0)	105 (4.1)
Moderately differentiated; Grade II	32,599 (26.1)	751 (44.8)	1,222 (47.5)
Poorly differentiated; Grade III	37,182 (29.8)	626 (37.4)	859 (33.4)
Undifferentiated; anaplastic; Grade IV	1,023 (0.8)	264 (15.8)	386 (15.0)
Unknown	44,254 (35.5)	–	
8th AJCC/UICC stage			
I	34,259 (27.5)	985 (58.8)	1,644 (63.9)
II	14,720 (11.8)	304 (18.1)	360 (14.0)
III	27,190 (21.8)	372 (22.2)	484 (18.8)
IV	48,619 (38.9)	14 (0.9)	84 (3.3)
Modified 8th stage			
I	33,803 (27.1)	994 (59.4)	1,614 (62.8)
II	28,048 (22.5)	535 (31.9)	704 (27.4)
III	25,647 (20.6)	139 (8.3)	188 (7.3)
IV	37,290 (29.8)	7 (0.4)	66 (2.5)
Treatment			
Surgery	44,194 (35.4)	1,675 (100)	2,572 (100)
No surgery	80,312 (64.4)	–	–
Unknown	282 (0.2)	–	–

### Modification of the TNM 8th Staging System

**Figure 1:**
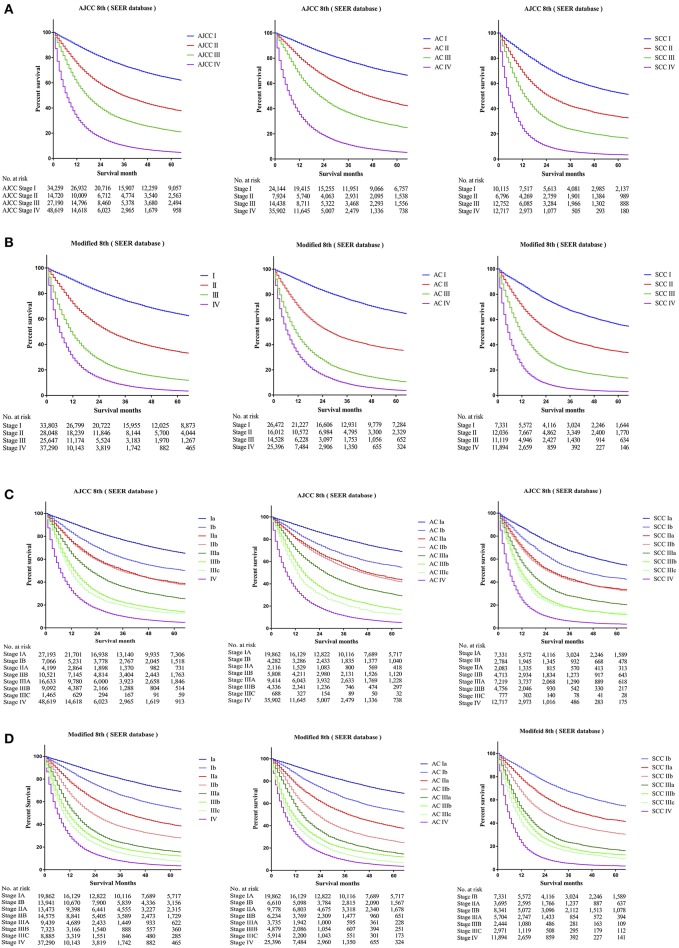
Kaplan-Meier survival curves for the patients and patients with adenocarcinoma or squamous cell carcinoma separately from the SEER cohort **(A)** using the 8th edition of the TNM staging system (I; II; III; IV), **(B)** the 8th edition of the TNM staging system (IA; IB; IIA; IIB; IIIA; IIIB; IIIC; IV), **(C)** the modified edition of the TNM staging system (I; II; III; IV), **(D)** the modified edition of the TNM staging system (IA; IB; IIA; IIB; IIIA; IIIB; IIIC; IV).

To identify whether patients' data from the SEER cohort for NSCLC was appropriate and accurate, we analyzed the survival of the patients in each stage by the Kaplan-Meier method based on the TNM 8th staging system. Overall, the 5-year CSS rates of stage I to IV patients, were 63.5, 39.2, 22.1, and 5.2%, respectively (Figure 1A). The hazard ratios (HR) for the comparisons between stage I and stage II, stage II and stage III, and stage III and stage IV were 0.467 [*p* < 0.0001, 95% confidence interval (CI), (0.4516 to 0.4830)], 0.6048 [*p* < 0.0001, 95% CI, (0.59–0.6201)], and 0.4973 [*p* < 0.0001, 95% CI, (0.4893–0.5054)], respectively (Table 2). Similarly, the HRs for the comparisons among sub-stages were statistically significant (Table 2), and the 5-year CSS rates of sub-stage IA to IV patients were 66.7, 51.3, 39.8, 38.9, 26.7, 15.0, 13.1, and 5.2%, respectively (Figure 1C). In contrast, we found that discrimination of survival curves of sub-stages was unsatisfactory in the current 8th TNM staging system, especially in the sub-stage of IIA and IIB (5-year CSS rate: 39.8% vs. 38.9%, HR = 0.9687, *p* = 0.3093) and IIIA and IIIB (5-year CSS rate: 15.0% vs. 13.1%, HR = 0.8931, *p* = 0.0005; Table 2).

**Table 2:** Cox proportional hazards regression model output for the 8th edition of the TNM staging system and modified staging system using the SEER cohort, FDZSH cohort, and PKUPH cohort.

**Stages compared**	**SEER cohort**				**FDZSH cohort**				**PKUPH cohort**			
	**Hazard ratio**		***P***		**Hazard ratio**		***P***		**Hazard ratio**		***P***	
	**8th edition**	**Modified edition**	**8th edition**	**Modified edition**	**8th edition**	**Modified edition**	**8th edition**	**Modified edition**	**8th edition**	**Modified edition**	**8th edition**	**Modified edition**
I to II	0.4670	0.4003	*p* < 0.0001	*p* < 0.0001	0.2685	0.2390	*p* < 0.0001	*p* < 0.0001	0.3429	0.2929	*p* < 0.0001	*p* < 0.0001
II to III	0.6048	0.4920	*p* < 0.0001	*p* < 0.0001	0.5791	0.5071	*p* < 0.0001	*p* < 0.0001	0.5424	0.4897	*p* < 0.0001	*p* < 0.0001
III to IV	0.4973	0.6286	*p* < 0.0001	*p* < 0.0001	1.0300	0.5202	*p* = 0.9310	*p* = 0.0841	0.6109	0.7504	*p* = 0.0029	*p* = 0.1313
IA to IB	0.5793	0.5739	*p* < 0.0001	*p* < 0.0001	0.3458	0.2295	*p* < 0.0001	*p* < 0.0001	1.4100	0.3941	*p* < 0.0001	*p* < 0.0001
IB to IIA	0.7243	0.6434	*p* < 0.0001	*p* < 0.0001	0.8165	0.6004	*p* = 0.3824	*p* = 0.0002	1.3060	0.5633	*p* = 0.0511	*p* < 0.0001
IIA to IIB	0.9687	0.7054	*p* = 0.3093	*p* < 0.0001	0.7300	0.8219	*p* = 0.2120	*p* = 0.0203	0.8350	0.6196	*p* = 0.2205	*p* = 0.0004
IIB to IIIA	0.7033	0.6796	*p* < 0.0001	*p* < 0.0001	0.6928	0.5494	*p* = 0.0010	*p* < 0.0001	1.0260	0.6808	*p* = 0.7789	*p* = 0.0134
IIIA to IIIB	0.6899	0.8246	*p* < 0.0001	*p* < 0.0001	0.5363	0.9640	*p* < 0.0001	*p* = 0.8702	1.1120	1.0150	*p* = 0.5286	*p* = 0.9631
IIIB to IIIC	0.8931	0.8530	*p* = 0.0005	*p* < 0.0001	0.5778	1.9250	*p* = 0.6931	*p* = 0.0969	2.9420	0.7121	*p* = 0.3651	*p* = 0.3473
IIIC to IV	0.6734	0.7461	*p* < 0.0001	*p* < 0.0001	2.4030	0.2802	*p* = 0.3929	*p* = 0.0094	0.3422	1.0500	*p* = 0.3223	*p* = 0.8508

*TNM, tumor, node, metastasis*.

Furthermore, we calculated the survival data of patients with adenocarcinoma or squamous cell carcinoma separately in the SEER cohort, which we had identified, for recursive partitioning and selection of the principal groupings, based on the hierarchical clustering model. Comparing with adenocarcinoma cases, patients with squamous cell carcinoma in the same sub-stage would usually have worse prognoses. For instance, stage Ib patients with adenocarcinoma may carry a similar prognosis as patients with squamous cell carcinoma in stage IA (5-year CSS rate: 56.3% vs. 56.1%, HR = 1.0160, *p* = 0.6091), and a prognosis between adenocarcinoma cases in stage IIb and squamous cell carcinoma cases in stage IB was similar as well (5-year CSS rate: 43.5% vs. 43.6%, HR = 0.9531, *p* = 0.1487). Similar results were also found among other sub-stages (Figure 1C).

**Figure 2:**
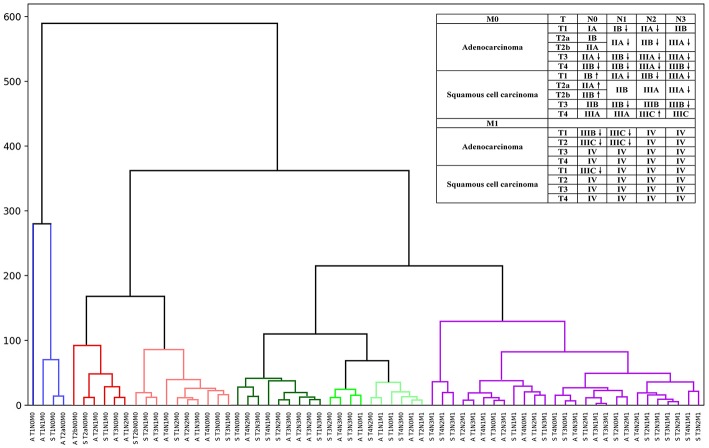
Hierarchical clustering model of the modified staging system using Kaplan-Meier curves and log-rank test statistics for recursive partitioning and selection of the principal groupings.

Thus, by maintaining the T, N, and M definitions of the current staging system, we regrouped the stages and sub-stages, and proposed a modified stage of the TNN staging system for NSCLC by the unsupervised learning result from the SEER cohort (Figure 2). Definitions of the 7 and 8th editions of AJCC/UICC TNM staging system and the modified staging system were showed in Figure 2.

The 5-year CSS rates of the modified stage I to IV, were 64.1, 34.5, 12.6, and 3.7%, and of the modified sub-stage Ia to IV, were 70.5, 55.0, 40.2, 29.1, 16.7, 12.7, 8.2, and 3.7%, respectively (Figures 1B,D). HRs for comparisons between the modified stage I and stage II, stage II and stage III, and stage III and stage IV, were 0.4003 [*p* < 0.0001, 95% CI, (0.3903 to 0.4105)], 0.492 [*p* < 0.0001, 95% CI, (0.4815 to 0.5027)], and 0.6286 [*p* < 0.0001, 95% CI, (0.6178 to 0.6395)], respectively (Table 2). Similar findings were also observed in comparisons among the modified sub-stage groups (Table 2). After the modification, the proportion of patients in stage Ia to IV is compared with the former, shows that the rationality and proportionality. In the modified staging system, more satisfactory discrimination of survival curves of sub-stages was shown, and similar results were detected in the FDUZH and PKUPH cohorts (Table 2).

### Comparison of Survival Outcomes Based on the Current and Modified 8th TNM Staging Systems

**Figure 3:**
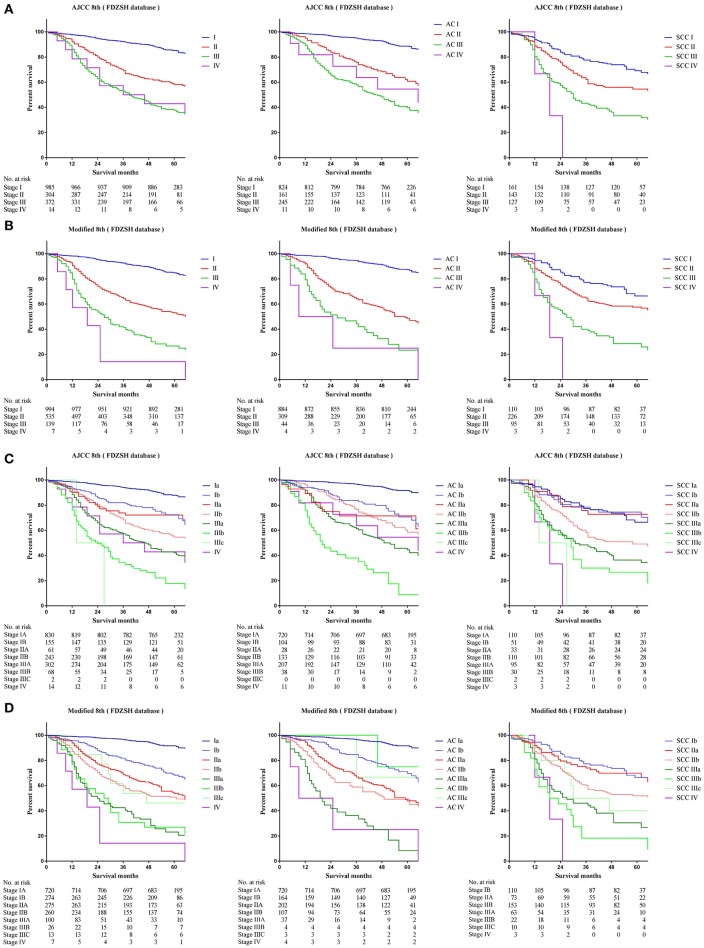
Kaplan-Meier survival curves for the patients and patients with adenocarcinoma or squamous cell carcinoma separately from the FDZSH cohort **(A)** using the 8th edition of the TNM staging system (I; II; III; IV), **(B)** the 8th edition of the TNM staging system (IA; IB; IIA; IIB; IIIA; IIIB; IIIC; IV), **(C)** the modified edition of the TNM staging system (I; II; III; IV), **(D)** the modified edition of the TNM staging system (IA; IB; IIA; IIB; IIIA; IIIB; IIIC; IV).

**Figure 4:**
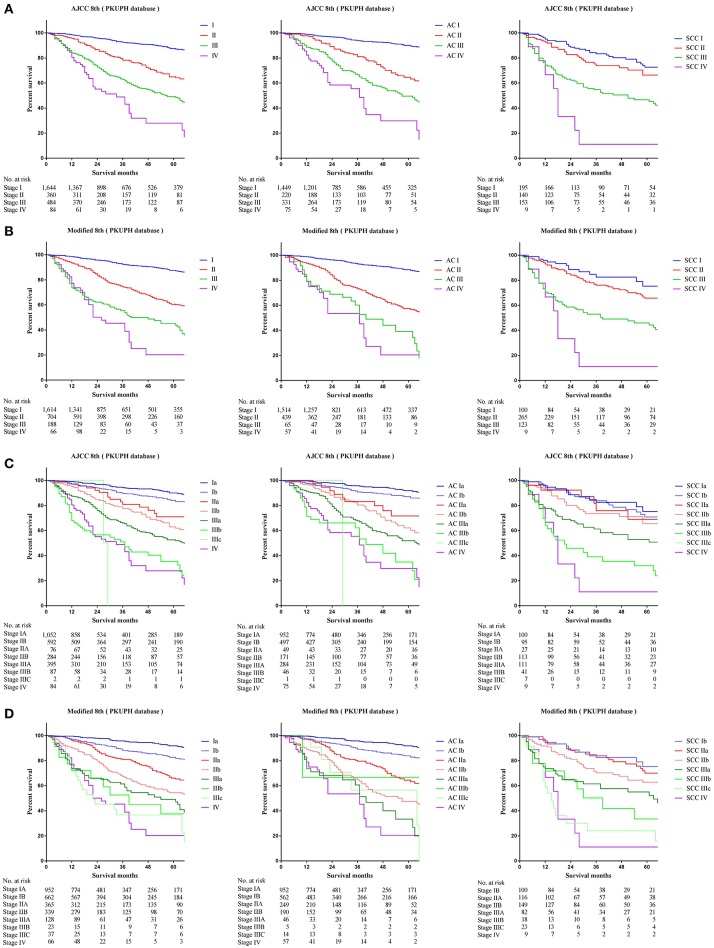
Kaplan-Meier survival curves for the patients and patients with adenocarcinoma or squamous cell carcinoma separately from the PKUPH cohort **(A)** using the 8th edition of the TNM staging system (I; II; III; IV), **(B)** the 8th edition of the TNM staging system (IA; IB; IIA; IIB; IIIA; IIIB; IIIC; IV), **(C)** the modified edition of the TNM staging system (I; II; III; IV), **(D)** the modified edition of the TNM staging system (IA; IB; IIA; IIB; IIIA; IIIB; IIIC; IV).

Comparing survival curves using the current TNM 8th staging system, the modified staging system indicated improved discrimination of survival curves for all cohorts from the SEER, FDZSH, and PKUPH databases (Figures 1, 3, 4). Accordingly, HRs for the comparisons between stage I and stage II, stage II and stage III, and stage III and stage IV improved substantially in the modified staging system (Table 2). However, according to the modified staging system, the 5-year CSS rates of stage I to IV patients from the FDZSH cohort were 84.7, 52.9, 26.7, and 14.3%, respectively, and patients from the PKUPH cohort were 87.6, 60.5, 45.0, and 20.2%, respectively (Figures 3B, 4B). Accordingly, HRs for the comparisons of stage I to stage II, stage II to stage III, and stage III to stage IV were 0.2390 [*p* < 0.0001, 95% CI, (0.1929–0.296)], 0.5071 [*p* < 0.0001, 95% CI, (0.3823–0.6725)], 0.5202 [*p* = 0.0841, 95% CI, (0.1859–1.4560)], and 0.2929 [*p* < 0.0001, 95% CI, (0.2335–0.3674)], 0.4897 [*p* < 0.0001, 95% CI, (0.3655–0.6562)], and 0.7504 [*p* = 0.1313, 95% CI, (0.4966–1.1340)], respectively (Table 2). The modified staging system showed superior discrimination and standardization of survival. Similar results of the 5-year CSS rates and HRs were also identified for the analyses among sub-stages, according to the current staging system, and modified staging system (Figures 3C,D, 4C,D; Table 2).

### Discrimination and Calibration Ability of the Current and Modified 8th TNM Staging Systems

C-indices of different staging systems for NSCLC are presented. The C-index of the modified staging system was significantly superior to that of the 8th staging system in all three cohorts. [SEER cohort: 0.722, 95% CI, (0.721–0.723) vs. 0.643, 95% CI, (0.640–0.647); FDZSH cohort: 0.720, 95% CI, (0.709–0.731) vs. 0.519, 95% CI, (0.450–0.586); and PKUPH cohort: 0.730, 95% CI, (0.705–0.735) vs. 0.728, 95% CI, (0.703–0.753)]. Similar results of sub-stages were also obtained for the SEER, FDZSH, and PKUPH cohorts [SEER cohort: 0.729, 95% CI, (0.727–0.731) vs. 0.657, 95% CI, (0.654–0.661); FDZSH cohort: 0.756, 95% CI, (0.731–0.781) *vs*. 0.644, 95% CI, (0.575–0.712); and PKUPH cohort: 0.749, 95% CI, (0.721–0.778) vs. 0.656, 95% CI, (0.628–0.685)]. As have been mentioned, the C-indices of modified staging systems showed better predictive ability and discrimination.

The calibration plots based on bootstrap resampling validation are illustrated in Supplemental Figures 1–4, which showed good agreement with the actual observations for 3-, and 5-year CSS. Thus, both in the discrimination test and calibration test of our modified staging system, the results showed superior predictive ability agreement with the actual observations for 3-, and 5-year CSS.

## Discussion

In our study, we used the unsupervised learning method by the deep learning to create a hierarchical clustering model for recursive partitioning and selection of the principal groupings, based on a large study cohort; therefore, the TNM stages with similar survival could be classified as the same group as much as possible. Based on the SEER database, we calculated and rebuilt a modified staging system according to the lung adenocarcinoma and squamous cell carcinoma data. Patients from the SEER database and FDZSH and PKUPH cohorts were then used to validate the reliability of our modified model, with results indicating that our modified staging system was more accurate in predicting the prognoses of patients with lung adenocarcinoma and squamous cell carcinoma. Likewise, the prognoses of different sub-stages with adenocarcinoma or squamous cell carcinoma differences were better discriminated in our modified staging system. We believe that our method using machine learning for modifying staging could have a positive impact on the effectiveness of prognostic estimation and benefit the staging systems of other cancers, and not only that of NSCLC. It seemed that survival prediction could be improved by machine learning.

For the last 40 years, the AJCC/UICC TNM Staging System of NSCLC has been regarded as the most precise model for the prognostic classification of patients with lung cancer and was well accepted in clinical practice. However, the new edition AJCC/UICC staging system may not be able to resolve the existing controversy regarding the differential survival and prognosis in the same stage of lung squamous cell carcinoma and lung adenocarcinoma and there are still problems of subjective ways for staging in AJCC/UICC TNM staging system. Demonstratively, the discrimination of prognoses of sub-stages, particularly in the sub-stage IIA and IIB, was unsatisfactory in the current TNM staging system, as shown in our analysis. A retrospective study in a large-scale Japanese cohort has identified significant differences in survivals between patients with adenocarcinoma and squamous cell carcinoma with 5-year survival rates of 78% in adenocarcinoma patients and 63% in squamous cell carcinoma patients (6). In particular, squamous cell carcinoma patients with stage I disease showed a significantly worse outcome than did adenocarcinoma patients (*p* < 0.0001), which indicated that different management and prognosis may exist in these patients. Evidence also suggests that lung adenocarcinoma and lung squamous cell carcinoma differ in the composition of genes and molecular characteristics (5, 16), such as *EGFR* gene mutations. It is noteworthy that outcomes are dynamic and change progressively with the new therapies, surgical, and radiotherapy techniques. The outcomes for patients treated in 2006, with only chemotherapy as the standard of care in advanced disease is not the same that in 2015 with targeted therapies or immunotherapy available. With the increased use of epidermal growth factor receptor-tyrosine kinase inhibitors, the survival rate of lung adenocarcinoma patients has improved substantially (17). However, few effective therapeutic targets for squamous cell carcinoma have been discovered (18–22).

As stated previously, the problems of subjective ways for staging and difference between lung adenocarcinoma patients and lung squamous cell carcinoma patients in survival cannot be ignored, like the current staging system of esophageal carcinoma, especially for surgeons. Therefore, we proposed to recalculation the TNM staging system for NSCLC, by considering survival differences of adenocarcinoma and squamous cell carcinoma and basing on the machine learning for the survival data for hierarchical clustering, which could have higher prognosis prediction and clinical guidance value for patients with NSCLC. Our results showed that the modified staging system was superior to the current TNM staging system in accuracy and reliability of predicting the prognosis of NSCLC.

Comparing the current TNM staging system, survival curves using the modified staging system were more sufficiently separated among sub-stages. In our new modified staging system, cases of patients with T1N1M0 adenocarcinoma will now be classified as stage IB, reflecting their better outcomes than those of cases involving tumors that remain in stage IIB. Similarly, the category T1N2M0, T2N1M0, and T3N0M0 of adenocarcinoma will move from IIB or IIIA to IIA. In addition, cases of T4N0M0 of adenocarcinoma, T3–4N1M0 of adenocarcinoma, and T2N2M0 of adenocarcinoma will now be classified as IIB, not IIIA, as was the case previously. In addition, T3–4N2M0 of adenocarcinoma and T2–4N3M0 of adenocarcinoma would also shift from IIIC to IIIB, IIIB to IIIA, and from IIIA to IIB. However, cases of T1–2N0M0 of squamous cell carcinoma would now move from IA to IB, IB to IIA, and from IIA to IIB, which reflect their worse outcome than that of cases involving tumors that remain in the original stage. Similarly, the results of the different survival rates of patients with lung adenocarcinoma and squamous cell carcinoma in the same TNM stage have been shown in several studies, which provides strong support for our modified staging system (7, 23).

In summary, compared with adenocarcinoma cases, squamous cell carcinoma cases would usually have been at a higher stage than adenocarcinoma cases of the same TNM. However, in the modified staging system, a worse outcome of squamous cell carcinoma than that of adenocarcinoma was noted. It is noticeable that these differences of survival and prognosis are often overlooked in clinical practice. Importantly, clinicians should undertake a comprehensive evaluation of patients with different histological data when they make clinical decisions, especially surgeons. Our results indicated that some cases of T1 with N0 disease but category M1 of both adenocarcinoma and squamous cell carcinoma also shifted from IV to IIIB or IIIC, and similarly, some cases of T2 with N2 disease but category M1 of adenocarcinoma moved from IV to IIIC, which was also noticeable. To a certain extent, we suggested that our results may indicate that compared with other M1 stage types, oligometastasis and M1a metastasis may have a better prognosis, which has been reported and improved in several studies these years (24, 25).

Inevitably, this study had several limitations. Our modified staging system was calculated and rebuilt based on the SEER database. Although this analysis included a large study cohort from the SEER database, which was population-based and provided detailed information regarding the patients, the prognosis of similar patients from other countries or ethnicities may be different from our cases. We regretted that we did not have access to the available data of driver oncogenes in the stage IV and data from systemic or radical therapies in early stage. In our study, a new unsupervised learning method was applied, which could provide a more accurate and reliable modified staging system, provided that a wide range of data could be analyzed. Second, the numbers of patients with stage III and stage IV in the FDZSH and PKUPH cohorts were small, because these patients did not receive surgery, which might have reduced the discrimination of the modified staging system to stage III and IV in these two cohorts, while C-indices still showed better predictive ability and discrimination of the modified staging system in stage III and IV. Importantly, we have to admit that the validation cohorts from Fudan and Peking university incorporate only patients who underwent surgical resection, whereas a staging system needs to be applicable to patients managed both surgically and non-surgically. Third, our modified staging system had instructional significance in the differentiation of prognoses; however, it is unclear whether this modified staging system would have better value in clinical practice. Thus, it is necessary to confirm our results using a large multi-institutional database and with multi-center large sample studies. Although our study proposed to reflect the differences of patients with NSCLC according to their different histological data, patients with large cell carcinoma were not considered, because of its low incidence, controversy of WHO classification, and unclear prognosis31. Finally, incorrect coding or erroneous data may have existed in the SEER database, and this source of error would be difficult to identify.

## Conclusion

The problems of staging and difference between lung adenocarcinoma and squamous cell carcinoma patients in survival should not be ignored when developing a more accurate discriminatory ability and prognostic performance in surgical practice. Differences of survival and more accurate and reliable prognosis in patients have been identified, which may refine prognostication over anatomic extent of TNM staging system. Staging system could be recalculated and improved by machine learning, which could have a positive impact on the effectiveness of prognostic estimation in the next edition TNM stage.

## Data Availability

The datasets generated for this study are available on request to the corresponding author.

## Author's Note

The American Association for Thoracic Surgery (AATS) 99th Annual Meeting, Toronto, Ontario, May, 2019.

## Author Contributions

ML and CZ: substantial contributions to the conception or design of the work, or the acquisition, analysis, or interpretation of data for the work. ML, CZ, and MF: drafting the work or revising it critically for important intellectual content. ML, QW, JW, XS, WJ, YS, XY, CZ, and MF: provide approval for publication of the content. ML, QW, CZ, and MF: agree to be accountable for all aspects of the work in ensuring that questions related to the accuracy or integrity of any part of the work are appropriately investigated and resolved.

### Conflict of Interest Statement

The authors declare that the research was conducted in the absence of any commercial or financial relationships that could be construed as a potential conflict of interest.

## Supplementary Material

Supplemental Figure 1 Calibration curves of predicting 3-year cancer-specific survival rates using the eighth edition of the TNM staging system for patients from **(A)** the SEER cohort (I; II; III; IV), **(B)** the SEER cohort IA; IB; IIA; IIB; IIIA; IIIB; IIIC; IV), **(C)** the FDZSH cohort (I; II; III; IV), **(D)** the FDZSH cohort (IA; IB; IIA; IIB; IIIA; IIIB; IIIC; IV), **(E)** the PKUPH cohort (I; II; III; IV), and **(F)** the PKUPH cohort (IA; IB; IIA; IIB; IIIA; IIIB; IIIC; IV).

Supplemental Figure 2 Calibration curves predicting 5-year cancer-specific survival rates using the 8th edition of the TNM staging system for patients from **(A)** the SEER cohort (I; II; III; IV), **(B)** the SEER cohort (IA; IB; IIA; IIB; IIIA; IIIB; IIIC; IV), **(C)** the FDZSH cohort (I; II; III; IV), **(D)** the FDZSH cohort (IA; IB; IIA; IIB; IIIA; IIIB; IIIC; IV), **(E)** the PKUPH cohort (I; II; III; IV), and **(F)** the PKUPH cohort (IA; IB; IIA; IIB; IIIA; IIIB; IIIC; IV).

Supplemental Figure 3 Calibration curves predicting 3-year cancer-specific survival rates using the modified staging system for patients from **(A)** the SEER cohort (I; II; III; IV), **(B)** the SEER cohort (IA; IB; IIA; IIB; IIIA; IIIB; IIIC; IV), **(C)** the FDZSH cohort (I; II; III; IV), **(D)** the FDZSH cohort (IA; IB; IIA; IIB; IIIA; IIIB; IIIC; IV), **(E)** the PKUPH cohort (I; II; III; IV), and **(F)** the PKUPH cohort (IA; IB; IIA; IIB; IIIA; IIIB; IIIC; IV).

Supplemental Figure 4 Calibration curves predicting 5-year cancer-specific survival rates using the modified staging system for patients from **(A)** the SEER cohort (I; II; III; IV), **(B)** the SEER cohort (IA; IB; IIA; IIB; IIIA; IIIB; IIIC; IV), **(C)** the FDZSH cohort (I; II; III; IV), **(D)** the FDZSH cohort (IA; IB; IIA; IIB; IIIA; IIIB; IIIC; IV), **(E)** the PKUPH cohort (I; II; III; IV), and **(F)** the PKUPH cohort (IA; IB; IIA; IIB; IIIA; IIIB; IIIC; IV).
