# Supplementary figures and images for: A Proposal to Reflect Survival Difference and Modify the Staging System for Lung Adenocarcinoma and Squamous Cell Carcinoma: Based on the Machine Learning
Source: Front Oncol. 2019 Aug 14;9:771. doi: 10.3389/fonc.2019.00771 (PMC6702456; doi:10.3389/fonc.2019.00771)

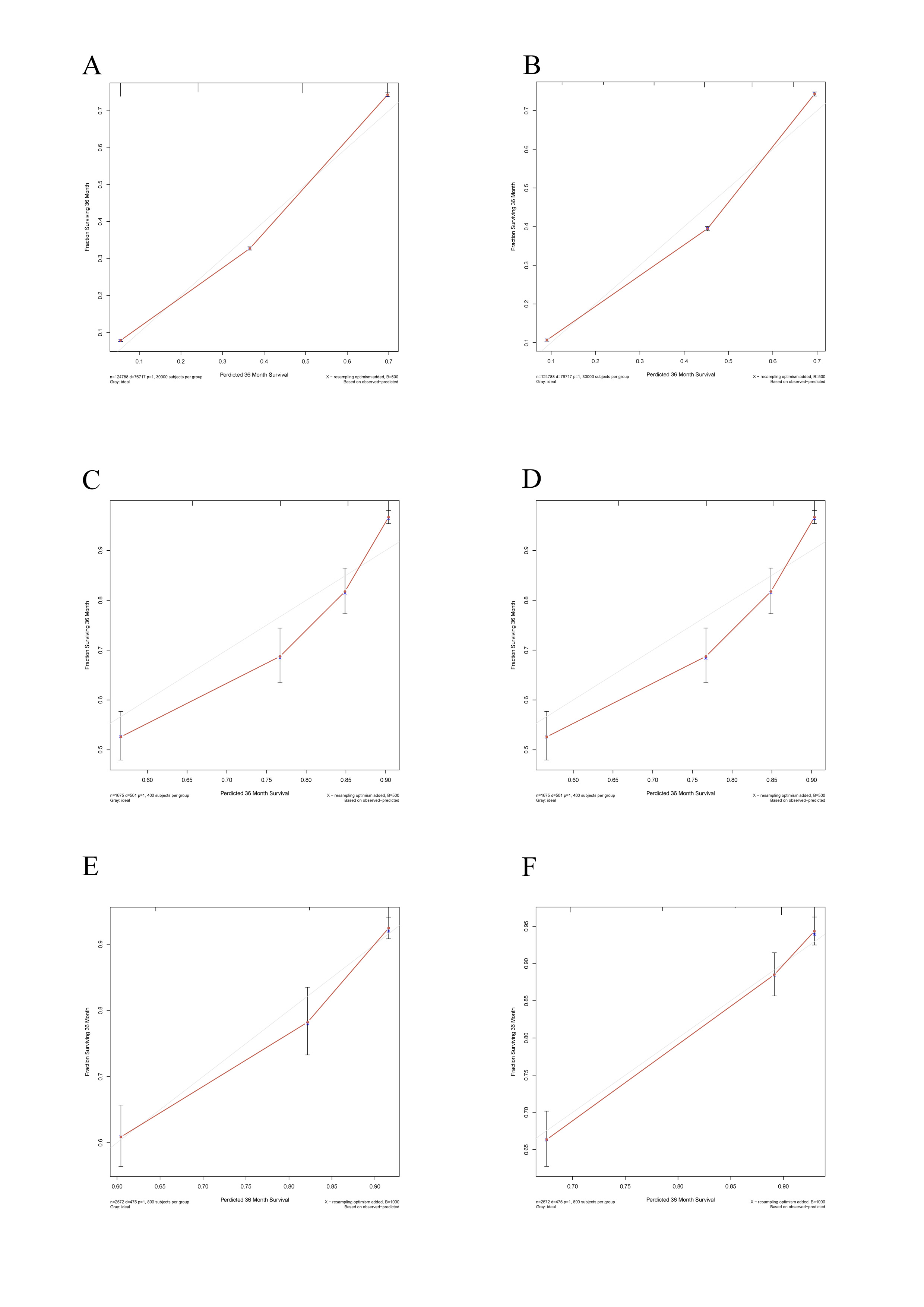

Supplement: Supplemental Figure 1 — Calibration curves of predicting 3-year cancer-specific survival rates using the eighth edition of the TNM staging system for patients from (A) the SEER cohort (I; II; III; IV), (B) the SEER cohort IA; IB; IIA; IIB; IIIA; IIIB; IIIC; IV), (C) the FDZSH cohort (I; II; III; IV), (D) the FDZSH cohort (IA; IB; IIA; IIB; IIIA; IIIB; IIIC; IV), (E) the PKUPH cohort (I; II; III; IV), and (F) the PKUPH cohort (IA; IB; IIA; IIB; IIIA; IIIB; IIIC; IV). [file Image_1.TIF]

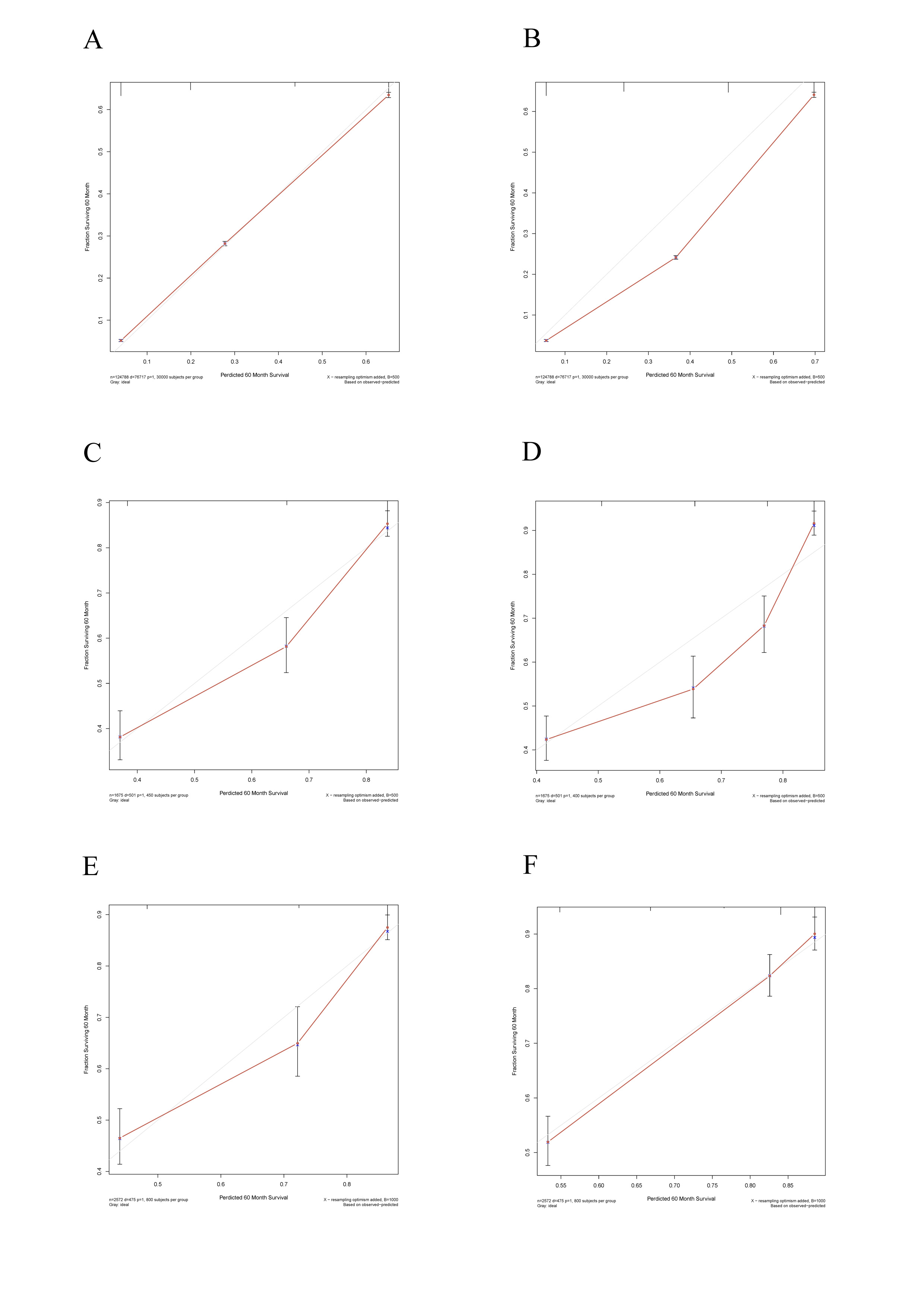

Supplement: Supplemental Figure 2 — Calibration curves predicting 5-year cancer-specific survival rates using the 8th edition of the TNM staging system for patients from (A) the SEER cohort (I; II; III; IV), (B) the SEER cohort (IA; IB; IIA; IIB; IIIA; IIIB; IIIC; IV), (C) the FDZSH cohort (I; II; III; IV), (D) the FDZSH cohort (IA; IB; IIA; IIB; IIIA; IIIB; IIIC; IV), (E) the PKUPH cohort (I; II; III; IV), and (F) the PKUPH cohort (IA; IB; IIA; IIB; IIIA; IIIB; IIIC; IV). [file Image_2.TIF]

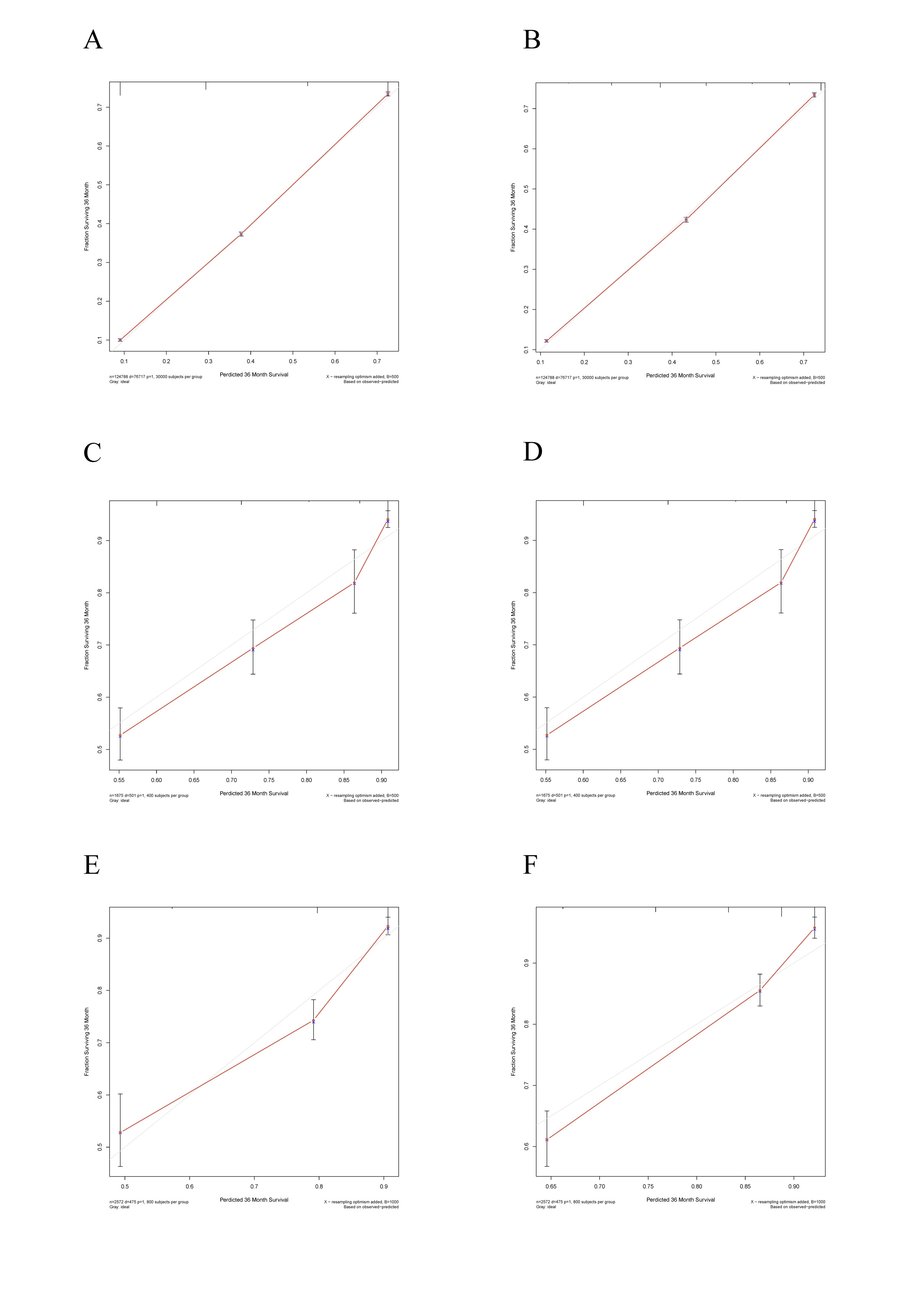

Supplement: Supplemental Figure 3 — Calibration curves predicting 3-year cancer-specific survival rates using the modified staging system for patients from (A) the SEER cohort (I; II; III; IV), (B) the SEER cohort (IA; IB; IIA; IIB; IIIA; IIIB; IIIC; IV), (C) the FDZSH cohort (I; II; III; IV), (D) the FDZSH cohort (IA; IB; IIA; IIB; IIIA; IIIB; IIIC; IV), (E) the PKUPH cohort (I; II; III; IV), and (F) the PKUPH cohort (IA; IB; IIA; IIB; IIIA; IIIB; IIIC; IV). [file Image_3.TIF]

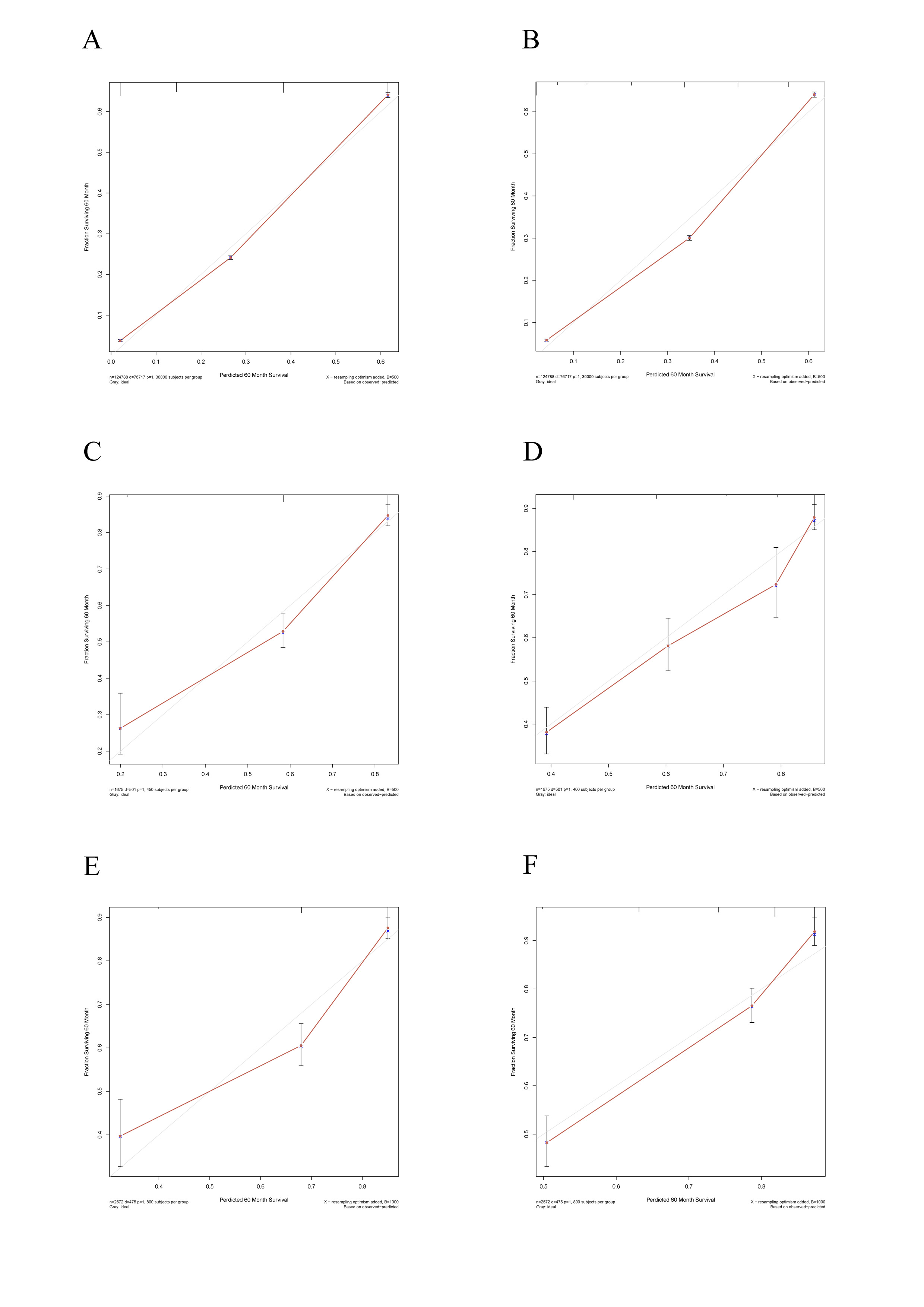

Supplement: Supplemental Figure 4 — Calibration curves predicting 5-year cancer-specific survival rates using the modified staging system for patients from (A) the SEER cohort (I; II; III; IV), (B) the SEER cohort (IA; IB; IIA; IIB; IIIA; IIIB; IIIC; IV), (C) the FDZSH cohort (I; II; III; IV), (D) the FDZSH cohort (IA; IB; IIA; IIB; IIIA; IIIB; IIIC; IV), (E) the PKUPH cohort (I; II; III; IV), and (F) the PKUPH cohort (IA; IB; IIA; IIB; IIIA; IIIB; IIIC; IV). [file Image_4.TIF]
